# Effects of ischaemic post-conditioning on eccentric exercise-induced muscle damage

**DOI:** 10.5114/biolsport.2024.129483

**Published:** 2023-09-21

**Authors:** Si Chen, Chuan Zhang

**Affiliations:** 1 School of Physical Education and Sport, Central China Normal University, Wuhan, China

**Keywords:** Resistance exercise, Remote effect, Delayed onset muscle soreness, Microvascular function, Muscle strength

## Abstract

Exercise-induced muscle damage (EIMD) is a common phenomenon resulting from high-intensity exercise that impairs subsequent performance. Ischaemic post-conditioning (IPOC) is a simple intervention that has been shown to reduce muscle damage after prolonged ischaemia, a condition mechanistically similar to EIMD. The purpose of this study was to determine whether IPOC could alleviate muscle damage after eccentric exercise. Thirty-two young male participants were randomized into either a sham (n = 16) or an IPOC (n = 16) intervention group. Biceps brachii muscle damage was induced by eccentric exercise, with IPOC or sham intervention applied on the dominant arm following exercise (3 cycles of 30 s ischaemia). Visual analogue scale (VAS) pain, arm circumference, muscle thickness, echo-intensity, and microvascular function (using near-infrared spectroscopy) were measured bilaterally at baseline, 24, 48, and 72 hours after eccentric exercise. Biceps curl one repetition maximum (1RM) was also measured. 1RM was higher for the IPOC group at 48 and 72 hours (both p < 0.05). On the dominant arm, VAS pain was lower at 72 hours for the IPOC group (p = 0.039). Muscle thickness was lower at all post-exercise time points for the IPOC group (all p < 0.05). VAS pain, echo-intensity, and arm circumference were elevated on the non-dominant arm in the sham group at 72 hours (all p < 0.05). These parameters all returned to the baseline level for the IPOC group at 72 hours (all p > 0.05IPOC could attenuate the decrease in strength, and alleviate EIMD with both local and remote effects after high-intensity exercise.

## INTRODUCTION

Exercise-induced muscle damage (EIMD) usually occurs after unaccustomed exercise, especially when there is a prominent eccentric component in the exercise performed [1]. The resultant damage is characterized by both immediate and prolonged symptoms following the exercise [2], and often manifests as delayed onset muscle soreness (DOMS), swelling and stiffness that could last for several days. Although EIMD is considered a mild injury, and is often regarded as a necessary process for post-exercise recovery [3], it may also greatly impair subsequent sports performance [4, 5]. Thus, when performance in subsequent tasks is important (as is the case with athletes or recreationally active individuals), prescribing effective and appropriate recovery techniques is crucial to preserve performance and avoid accumulative injury.

Previously studied recovery methods, including massage, cold water immersion, electrical stimulation and compression garments, have demonstrated effectiveness with the goal of alleviating EIMD and DOMS [6–8]. However, these methods are less feasible for athletes as they require assistance, additional equipment and/or space. Given the challenges associated with the previously mentioned methods, it is necessary to explore effective, affordable, and time-efficient methods of recovery that can be performed by a single individual.

Recent research using multiple short cycles of ischaemia and reperfusion as an intervention has gained considerable focus and is now highly valued in the medical and sports fields [9, 10]. Ischaemic preconditioning (IPC), which was originally applied before heart surgery to reduce cardiac muscle damage after long-term ischaemia [11], has been applied to athletic populations to alleviate fatigue and improve performance [12, 13]. Importantly, recent studies have shown that both acute and repetitive IPC are effective for reducing EIMD and DOMS [14, 15]. Similar in concept, ischaemic post-conditioning (IPOC) is applied acutely after tissue-damaging physiological conditions, and also uses multiple short-term ischaemia-reperfusion cycles. IPOC has been shown to produce protective effects on prolonged ischaemia-induced muscle damage through similar mechanisms to that of IPC [16]. IPOC may also have effects on ischaemic limbs that have not directly received IPOC treatment, otherwise known as remote effects [17]. However, the application of IPOC in sports has been scarce, and whether IPOC could reduce EIMD and DOMS remains to be elucidated.

There are two noteworthy differences between IPOC and IPC. The first is that IPOC is applied in the early stages of recovery rather than prior to ischaemia, and the second is that it takes much less time to perform (~3 minutes for IPOC vs. 25–35 minutes for IPC) than IPC. The application of IPOC can be achieved using a simple sphygmomanometer and does not include active exercise or additional energy consumption, making it a passive strategy for sport implementation. From a mechanistic perspective, EIMD and ischaemia-reperfusion injury influence ion homeostasis, cell acidosis, and immune responses similarly [14]. For this reason, it is plausible to suggest that IPOC may also alleviate EIMD and DOMS. However, to the best of our knowledge, no human studies have directly examined the influence of IPOC on EIMD and DOMS.

Therefore, the first aim of this study was to evaluate whether IPOC has protective effects against EIMD and DOMS, and whether a remote effect exists. Additionally, because IPOC is known to have vascular protective effects [18], and EIMD is often associated with impaired vascular function [19], the second aim of the study was to determine whether the potential beneficial effects of IPOC against EIMD and DOMS might be mediated through the improvement of vascular function.

## MATERIALS AND METHODS

### Participants

Thirty-two young healthy males, without any known cardiovascular or musculoskeletal disease and injury, between the ages of 18 and 30, were recruited for this study. The inclusion criteria were 1) not having performed any resistance training routine for the past six months, and 2) not actively engaged in any other sports for the past six months. We targeted this group to avoid the potential influence of gender and training status on the study variables. All participants reported being right-hand dominant. The participants were randomly assigned to either an IPOC intervention group (n = 16) or a sham group (n = 16).

### Ethics

This study was approved by an institutional review board (approval No. CCNU-IRB-202212006) and was conducted in accordance with the Declaration of Helsinki. All participants received an explanation of the study aims and methods, and they signed a consent form prior to data collection.

### Experimental design and procedure

**FIG. 1:**
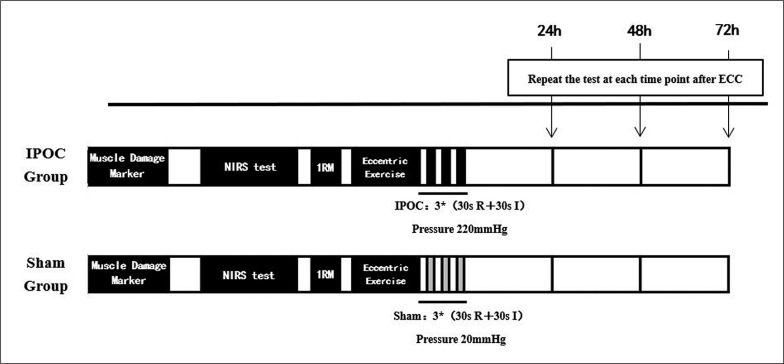
Experimental design. IPOC, ischemic postconditioning; EIMD, exercise-induced muscle damage; R, tourniquet release; I, tourniquet inflation.

This study used a parallel group design, with the between-group factor being intervention type (IPOC vs. sham) and the within-group factor being time (baseline, 24, 48 and 72 hours after exercise). The experiment was conducted over four days. Each participant completed the same procedures at the same time of day to remove any circadian influences on exercise effort, vascular function, and subjective feelings. Muscle damage was induced by performing eccentric exercise using a barbell and plates. Immediately after eccentric exercise, the ischaemic intervention or sham control intervention was applied to participants. Before and 24, 48 and 72 hours after eccentric exercise and intervention, EIMD markers including the visual analogue scale (VAS) for pain, muscle thickness, arm circumference, and echo intensity of the biceps brachii were measured bilaterally. Additionally, bilateral microvascular function by near-infrared spectroscopy (NIRS) and elbow flexion 1RM were measured (Figure 1).

### Eccentric exercise

The biceps curl exercise was used to induce muscle damage and DOMS bilaterally on the biceps brachii. Resistance was applied using a barbell and plates at 80% of each participant’s one repetition maximum (1RM) as the exercise intensity. The exercise consisted of three sets of ten repetitions with a one-minute rest period between sets. Participants were instructed to assume and maintain an erect position, with backs against a wall and elbows at their sides throughout the repetitions. Participants completed a 4 second eccentric (lowering portion) curl through a full range of motion (from 50° to 180°), while two assistants lifted the barbell back to 180° (from 50°) in a coordinated fashion [14]. This was adhered to as a way to ensure that only eccentric repetitions contributed to EIMD.

The ischaemic and sham control interventions were applied to the right biceps brachii muscle of each participant. All tests were performed bilaterally, with the exception of 1RM. All tests were conducted in the same laboratory throughout the experiment, with a laboratory temperature set at 24–25 degrees Celsius. The same tester conducted all the tests throughout the experiment to eliminate inter-tester variance.

### IPOC or sham intervention

The post-exercise intervention was applied 30 s after finishing the last set using a tourniquet. The tourniquet was placed on the proximal humerus of the dominant arm. The non-dominant arm was also tested to determine whether a remote effect for IPOC exists. For the IPOC intervention group, the tourniquet was inflated to 220 mmHg for 30 s to achieve ischaemia. The 30 s inflation and deflation was repeated for 3 cycles, which has been previously performed [18]. For the sham group, the pressure was inflated to 20 mmHg for 30 s and the cycle was repeated for 3 cycles. By applying a minimal pressure tourniquet in the sham condition, we hoped to control the potential placebo effect that may influence the experimental results. Since all participants were right-side dominant, the intervention was performed on the right arm of participants. The total intervention time was 3 minutes.

### VAS pain

Perceived pain intensity was measured using a 100 mm VAS scale with 0 indicating no pain and 100 indicating the most excruciating pain imaginable. Participants were instructed to draw a vertical mark on this scale representing their current level of pain.

### Arm circumference

Arm circumference was taken as a surrogate for muscle swelling, and measured using a flexible tape [14]. The assessment was conducted at 60% of the length between the acromial process and the lateral epicondyle (closer to the distal end of the upper arm). The locations of the measurement were marked using a permanent marker to ensure consistent measurement location throughout the experiment. Each participant’s arm was measured three times, and the average measure of the three was recorded as arm circumference.

### Muscle thickness and echo-intensity

Muscle thickness and echo-intensity were measured using a B-mode ultrasound (Logiq E, GE Healthcare China, Wuhan, China). The musculoskeletal ultrasound preset was used throughout the test while all time gains were set to a neutral position. An 8–12 MHz multi-frequency linear transducer was used. Scan depth was kept at 4 cm. Gains were set to 58 db while transducer frequency was set to 8 MHz. Other settings remained unaltered [20]. The location of the ultrasound measurement was consistent with the previously described location of the assessment of arm circumference (60% of the distance between the acromial process and the lateral epicondyle) and were marked as previously described to ensure consistent measurement. The measurement was taken longitudinally and repeated twice. The obtained images were then processed using ImageJ (Version 1.37, National Institute of Health, Bethesda, MD, USA) software. Muscle thickness was first determined at the centre of the image, and echo-intensity was then determined by manually tracing the biceps brachii area, before calculating the mean pixel intensity.

### 1RM test

1RM was assessed by testing elbow flexion strength with a barbell weighing 5 kg, and additional weight was added with 0.5 kg accuracy. The participant performed warm-up exercise consisting of 4–5 bilateral elbow flexions with the barbell. For the actual 1RM test, the participant performed elbow flexion from 180^o^ (elbow fully extended) to 50^o^ with the initial weight set to an estimated 80% 1RM. Weight was then adjusted after consulting the participant. Sufficient rest time was given between two consecutive attempts. The maximal weight that the participant was able to lift throughout the 180^o^ to 50^o^ range of motion was considered as 1RM [14]. All 1RM trials were completed within 5 attempts.

### Microvascular function

Microvascular function was measured using near infrared spectroscopy (NIRS) [21]. A continuous wavelength NIRS probe (PortaLite, Artinis Medical Systems B.V., Einsteinweg, The Netherlands) was placed on the muscle belly and secured using double-sided tape and pre-wrap. The location of the NIRS probe placement was consistent with the previously described location of the assessment of arm circumference (60% of the distance between the acromial process and the lateral epicondyle). The location of the probe was traced using a permanent marker to ensure a consistent measurement location throughout the experiment. A tourniquet was placed proximally to the NIRS probe and connected to a custom-made rapid cuff inflation device, which can inflate a cuff to a pre-determined pressure within 0.5 s. The participant lay quietly on a bed for at least 5 minutes before the test started. The tourniquet was then rapidly inflated to 220–250 mmHg for 5 minutes, and then rapidly deflated. The NIRS signal was continuously monitored throughout the test and for at least 2 minutes following tourniquet release.

NIRS sensors use spatially resolved spectroscopy method to obtain the tissue saturation index (TSI) for local muscle oxygenation measurement. This reflects the dynamic balance between oxygen delivery and oxygen extraction. The TSI slope of change within the first 10 s of tourniquet release (TSI10) was calculated as an indicator for skeletal muscle microvascular function [22, 23]. The acquired data were processed using a custom-written MATLAB (version 2020b, MathWorks, Natick, MA, USA) script.

### Statistics

All data were analysed using SPSS 27.0 software (IBM Corp., Armonk, NY, USA). Results were expressed as means ± standard deviations (mean ± SD). The Shapiro-Wilk method was used to test the distribution of data. Data that followed a normal distribution were analysed using a two-way ANOVA with group (IPOC or sham) representing the between-group factor while time (baseline, 24, 48 and 72 hours after exercise) represented the within-group repeated factor.

Data that violated the assumption of normality were analysed using nonparametric tests (independent samples and related samples) to assess between-group and within-group differences. All significance levels were set at the 0.05 level. Where appropriate, Cohen’s d was calculated to determine effect size, with 0.2, 0.5 and 0.8 representing small, medium and large effects [24].

## RESULTS

Table 1 summarizes physical characteristics for all participants. No significant between-group differences were detected (all p > 0.05). Baseline 1 RM was similar between the IPOC and sham groups (p = 0.119). Furthermore, no significant differences in VAS pain, echo-intensity, and arm circumference were observed between the IPOC and sham groups for either arm (all p > 0.05). However, participants in the sham group exhibited greater muscle thickness in the non-dominant arm compared to those in the IPOC group (p = 0.041). Due to this identified difference, baseline non-dominant arm muscle thickness was used as a covariate in further analysis.

**TABLE 1:** Physical characteristics for participants. Sham, group that received sham treatment; IPOC, group that received ischemic postconditioning treatment; ATT, adipose tissue thickness.

	Sham (N = 16)	IPOC (N = 16)
Age (Years)	21.8 ± 2.8	21.4 ± 2.9
Height (Cm)	174.5 ± 4.4	173.9 ± 7.2
Weight (Kg)	65.4 ± 8.16	66.3 ± 10.4
Right Biceps Att (Cm)	0.44 ± 0.14	0.40 ± 0.12
Left Biceps Att (Cm)	0.42 ± 0.15	0.43 ± 0.11

The IPOC group demonstrated better 1RM performances at 48 and 72 hours (p = 0.032, 0.047; d = 0.527, 0.506) following eccentric exercise when compared to the sham group.

**FIG. 2:**
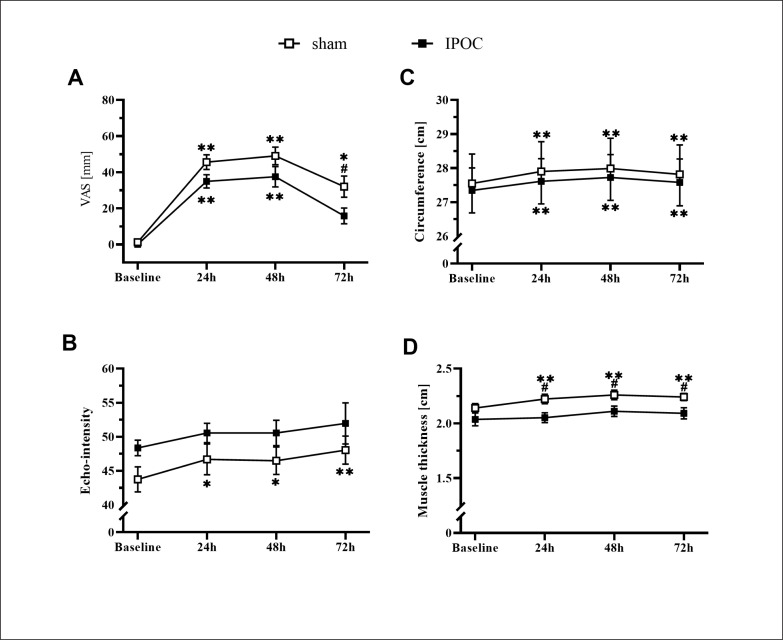
Results for the dominant arm for visual analog scale (VAS) pain, arm circumference, echo-intensity and muscle thickness at baseline and 24, 48 and 72 hours after eccentric exercise. *p <0.05, **p<0.01 compared to baseline; # significant between groups difference.

Pain was lower in the dominant arm at 72 hours in the IPOC group compared to the sham group (p = 0.039; d = 0.790). No differences were observed in echo-intensity for the IPOC group at all post-exercise time points when compared to baseline (p = 0.318 for repeated ANOVA) (Figure 2). Echo-intensity was higher compared to baseline at all post-exercise time points for the sham group (p = 0.018, 0.044 and < 0.001; d = 0.356, 0.352 and 0.549, respectively for 24, 48 and 72 hours). Muscle thickness was greater in the sham group compared to the IPOC group at each post-exercise time point (p = 0.012, 0.027 and 0.021; d = 0.944, 0.277 and 0.859, respectively for 24, 48 and 72 hours). There was a trend for TSI10 to increase following eccentric exercise for both the IPOC and the sham groups. At 72 hours, the IPOC group experienced a decrease in TSI10, while the sham group remained elevated; however, none of these between-group or within-group measures reached statistical significance (Figure 4A).

**FIG. 3:**
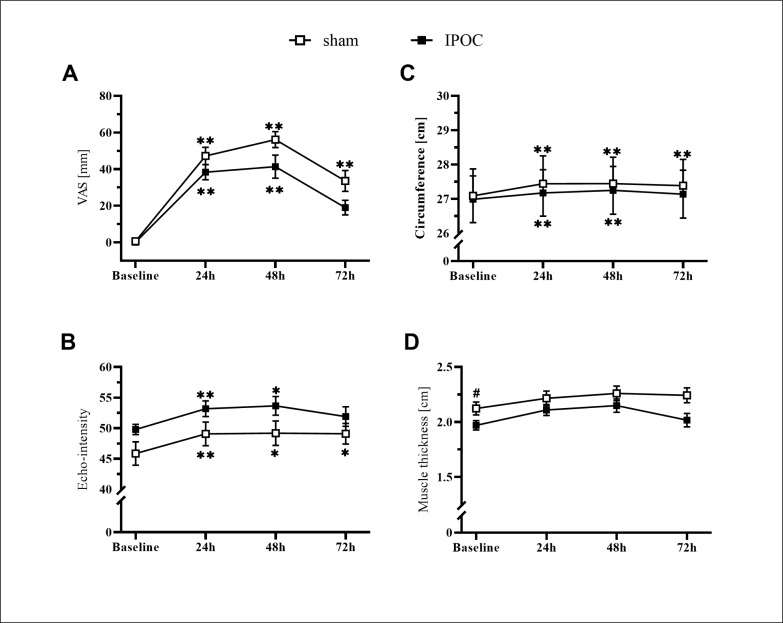
Results for the non-dominant arm for visual analog scale (VAS) pain, arm circumference, echo-intensity and muscle thickness at baseline and 24, 48 and 72 hours after eccentric exercise. *p <0.05, **p<0.01 compared to baseline; # significant between groups difference.

**FIG. 4:**
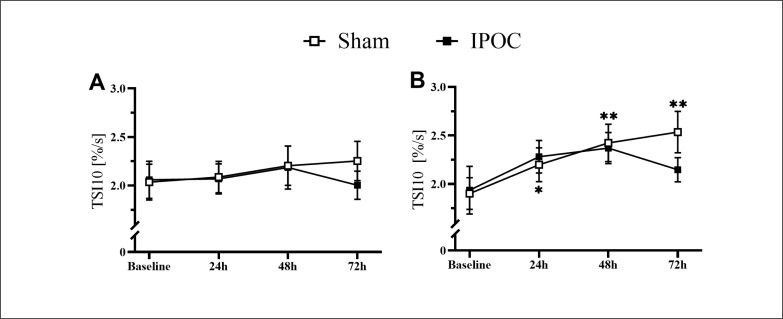
Microvascular function changes as measured by tissue saturation index (TSI) 10 seconds slope following tourniquet release (TSI10) on the dominant (A) and non-dominant side (B) for both groups. *p <0.05, **p<0.01 compared to baseline.

Non-dominant arm pain, echo-intensity, and arm circumference were elevated in the sham group at 72 hours compared to baseline (p = 0.006, 0.014 and 0.003; d = 2.096, 0.461 and 0.100, respectively). These markers for muscle damage all returned to the baseline level for the IPOC group at 72 hours (p = 0.100, 0.230 and 0.097; d = 1.722, 0.420 and 0.056, respectively; Figure 3). The sham group showed increased values in TSI10 compared to baseline at every post-exercise time point (p = 0.024, < 0.001 and 0.004; d = 0.482, 0.707 and 0.914, respectively for 24, 48 and 72 hours). No change in TSI10 was detected in the IPOC group compared to baseline (p = 0.161, 0.091 and 0.345; d = 0.346, 0.508 and 0.222, respectively for 24, 48 and 72 hours; Figure 4B).

## DISCUSSION

Muscle damage after high-intensity exercise is a common and well-documented phenomenon [1, 2]. This study adds to the literature by exploring the effectiveness of IPOC in alleviating muscle damage and associated symptoms after eccentric exercise. We found that IPOC can accelerate the recovery of EIMD markers with both local and remote effects, and also mitigate the strength decline following eccentric exercise. These results corroborate findings from previously published studies that examined the effects of IPC on EIMD [14, 15]. While the protective effects of IPOC seen in the current study seem to be smaller than those of IPC, the shorter intervention time and the ability to apply intervention immediately after exercise make IPOC a viable, efficient alternative for reducing DOMS and EIMD.

The mechanisms through which IPOC improves EIMD markers are complex and remain to be fully elucidated. Previous studies investigating muscle damage caused by ischaemia-reperfusion injury suggest that IPOC may act on adenosine and ATP-sensitive potassium channels in the post-processing phase after ischaemia [25, 26]. An increase in adenosine levels and opening of ATP-sensitive potassium channels can promote vasodilation, thus regulating the balance of oxygen and metabolic substrate transport during exercise [27]. Consequently, one can infer that the alleviation of muscle damage following eccentric exercise might also be related to these mechanisms. On the other hand, the increased production of nitric oxide (NO) synthase may also contribute to the protective effects of IPOC against EIMD [28]. NO release as a result of IPOC application activates muscle fibre satellite cells, which then enter the proliferation cycle [29, 30]. NO also has anti-inflammatory functions, which can help alleviate the inflammatory response and reduce the muscle damage response after high-intensity training [31]. Additionally, the remote effect of IPOC has been confirmed in various tissue organs. For instance, remote IPOC of the femoral artery can alleviate brain damage resulting from focal cerebral ischaemia [32], an observation similar to the remote protective effect documented in this study. Previous studies have demonstrated that the mechanisms underlying IPOC improvements in physiological conditions could be related to factors such as neural plasticity and synapse formation, and may be regulated by the hypoxia-inducible factor 1α signal, as well as through the activation of adenosine receptors [33, 34].

We sought to investigate the role of vascular function in explaining the protective effect of IPOC against EIMD. Many studies have shown that microcirculatory function is impaired after eccentric exercise [35, 36]. In this study, the increase in TSI10 after muscle damage caused by eccentric exercise does not necessarily indicate an improvement in microcirculation function. Our results are consistent with previous studies which indicate that microvascular perfusion is increased after EIMD [37, 38]. The mechanism for this change is not completely understood, but human studies on arterial function may shed some light. Previous studies have shown that, while flow-mediated dilation decreased after eccentric exercise, resting baseline diameter may actually increase [19, 39]. This is likely the result of an increase in the surface area for exchange of substrates that needs to pass through the basement membrane after eccentric exercise [40], causing macrophages, neutrophils, and other repair cytokines to accumulate around the damaged area, leading to an increase in blood flow [38]. In animal models, capillary diameter was increased following eccentric exercise [35]. As a result, the elevation in TSI10 could stem from an increased vessel diameter due to muscle damage, rather than enhanced microvascular reactivity, an inference requiring further study for confirmation. The trend for TSI10 to increase following eccentric exercise is more evident in the non-dominant arm, and IPOC seems to exert a better vascular protective effect in this arm as well. This finding could be related to the present study design. The same eccentric exercise load was applied to both upper limbs of the subjects in our biceps curl paradigm, while the intervention was only done on the dominant arm. It is possible that the non-dominant arm showed more significant vascular damage under the same eccentric exercise load intensity due to weaker strength. Therefore, the effects of IPOC intervention are more prominent than on the dominant arm. However, this inference remains to be confirmed with further studies.

There are practical implications of this study. The results provide scientific evidence to support the use of an affordable and time-efficient means to reduce EIMD and DOMS after high-intensity exercise. Since exercisers can apply IPOC with just a simple sphygmomanometer without the need for additional assistance, it could serve as an ideal recovery agent. Additionally, since IPOC is applied at the early recovery phase rather than before exercise (like IPC), IPOC could be used in situations where EIMD is not anticipated prior to the workout session. However, when compared to the effects of IPC reported in the literature [14], the magnitude of protection seems to be less for IPOC. Important to note, this finding was not directly demonstrated in this study. Therefore, individuals who intend to utilize IPOC and reap its purported benefits should be aware of this potential limitation of the technique.

The limitations of this study must be addressed. Firstly, the study participants were without recent resistance training experience; thus it is uncertain whether IPOC would exert similar protective effects on individuals who frequently engage in resistance training. This is certainly a topic warranting further examination. Second, as gender has previously been shown to influence the EIMD response [41], the results of this study cannot be applied to women without a dedicated study design. Thirdly, while we have proposed potential explanations, the cause of the post-exercise increased TSI10 in the non-dominant arm remains elusive, necessitating future mechanistic research to shed light on the underlying mechanisms. Fourthly, multiple IPOC protocols exist for consideration. The current study employed the three 30-second cycles of IPOC, a protocol commonly mentioned in the literature [18, 42, 43]. Previous studies have investigated the effects of different IPOC protocols on ischaemic-reperfusion injury [44]. Changing the number of cycles or ischaemic duration may alter the protective effects of IPOC against EIMD, a possibility warranting further research.

## CONCLUSIONS

In conclusion, IPOC could attenuate the decrease in strength, and alleviate EIMD with both local and remote effects after high-intensity exercise. The protective effect might potentially be associated with improvements in vascular function. Nonetheless, additional research is required to elucidate the underlying mechanisms. Further investigations are necessary to determine whether IPOC confers similar protective effects in females and seasoned exercisers. Furthermore, the influence of different IPOC protocols on EIMD necessitates more in-depth exploration.
